# Pediatric Brain Abscesses, Epidural Empyemas, and Subdural Empyemas Associated with *Streptococcus* Species — United States, January 2016–August 2022

**DOI:** 10.15585/mmwr.mm7137a2

**Published:** 2022-09-16

**Authors:** Emma K. Accorsi, Sopio Chochua, Heidi L. Moline, Matt Hall, Adam L. Hersh, Samir S. Shah, Amadea Britton, Paulina A. Hawkins, Wei Xing, Jennifer Onukwube Okaro, Lindsay Zielinski, Lesley McGee, Stephanie Schrag, Adam L. Cohen

**Affiliations:** ^1^Division of Bacterial Diseases, National Center for Immunization and Respiratory Diseases, CDC; ^2^Epidemic Intelligence Service, CDC; ^3^Children’s Hospital Association, Lenexa, Kansas; ^4^Department of Pediatrics, Division of Infectious Diseases, University of Utah, Salt Lake City, Utah; ^5^Cincinnati Children’s Hospital Medical Center, Cincinnati, Ohio.

In May 2022, CDC learned of three children in California hospitalized concurrently for brain abscess, epidural empyema, or subdural empyema caused by *Streptococcus intermedius*. Discussions with clinicians in multiple states raised concerns about a possible increase in pediatric intracranial infections, particularly those caused by *Streptococcus* bacteria, during the past year and the possible contributing role of SARS-CoV-2 infection (*1*). Pediatric bacterial brain abscesses, epidural empyemas, and subdural empyemas, rare complications of respiratory infections and sinusitis, are often caused by *Streptococcus* species but might also be polymicrobial or caused by other genera, such as *Staphylococcus*. On June 9, CDC asked clinicians and health departments to report possible cases of these conditions and to submit clinical specimens for laboratory testing. Through collaboration with the Children’s Hospital Association (CHA), CDC analyzed nationally representative pediatric hospitalizations for brain abscess and empyema. Hospitalizations declined after the onset of the COVID-19 pandemic in March 2020, increased during summer 2021 to a peak in March 2022, and then declined to baseline levels. After the increase in summer 2021, no evidence of higher levels of intensive care unit (ICU) admission, mortality, genetic relatedness of isolates from different patients, or increased antimicrobial resistance of isolates was observed. The peak in cases in March 2022 was consistent with historical seasonal fluctuations observed since 2016. Based on these findings, initial reports from clinicians (*1*) are consistent with seasonal fluctuations and a redistribution of cases over time during the COVID-19 pandemic. CDC will continue to work with investigation partners to monitor ongoing trends in pediatric brain abscesses and empyemas.

Two data sources were analyzed: 1) pediatric hospitalizations for brain abscesses, epidural empyemas, and subdural empyemas reported to CHA’s Pediatric Health Information System (PHIS) and 2) cases reported to CDC in response to a national call for cases. With CHA, CDC examined hospitalizations at 40 tertiary referral children’s hospitals across the United States that consistently reported data to PHIS during January 1, 2016–May 31, 2022 (the most recent data available when the analysis was performed). All inpatient encounters from patients aged ≤18 years with a primary or secondary discharge diagnosis of *International Classification of Diseases, Tenth Revision, Clinical Modification* code G06.0 (intracranial abscess and granuloma) or G06.2 (extradural and subdural abscess, unspecified) during the study period were included. Concurrent COVID-19 diagnosis was defined as having *International Classification of Diseases, Tenth Revision* codes U07.1 or B97.29 on the discharge diagnosis list. Medical complexity was classified according to the Pediatric Medical Complexity Algorithm (*2*).

In CDC’s national call for cases, a case was defined as the diagnosis of brain abscess, epidural empyema, or subdural empyema in a person aged ≤18 years without a previous neurosurgical procedure or history of head trauma, hospitalized on or after June 1, 2021, irrespective of etiology. The call for cases was shared with health departments and two provider listservs.* Reports received after August 10, 2022, were excluded. Available *Streptococcus* specimens isolated from a brain abscess, epidural empyema, subdural empyema, blood, or cerebrospinal fluid were collected for antimicrobial susceptibility testing and whole-genome sequencing at CDC's *Streptococcus* reference laboratory to identify microbiological features shared among cases. Genomic sequences were generated with an Illumina Miseq (*3*) instrument, and single-nucleotide polymorphisms (SNPs) were identified for core genomes employing kSNP3.0 with k-mer size of 19 (*4*). Pairwise comparisons were generated employing Mega7 (*5*). Minimal inhibitory concentrations (MICs) were determined by broth microdilution methods according to the Clinical and Laboratory Standards Institute (*6*). The agar diffusion gradient method (Etest, bioMérieux) was used for isolates that did not grow in broth. Analyses were conducted using SAS (version 9.4; SAS Institute) or R (version 4.0.3; R Foundation) with R Studio (version 1.3.1093; RStudio, PBC).This study was reviewed by CDC and was conducted consistent with federal law and CDC policy.^†^

## Cases Identified Through CHA’s PHIS Database

During January 2016–May 2022, a total of 3,078 cases of pediatric brain abscesses, epidural empyemas, or subdural empyemas were identified from the PHIS database, ranging from 20 to 68 cases per month (median = 38; IQR = 32–48) (Figure). Beginning in April 2020, case counts were below the median for 15 months, the longest such interval during the analysis period. Starting in summer 2021, cases increased and peaked in March 2022, representing the longest interval with case counts above the median, before declining in April 2022. During these two periods, 184 fewer and 177 more cases occurred, respectively, than would have, if each month had had the median number of cases. Since 2016, peaks in cases have often occurred around March, with similarly sized peaks observed in March 2017 and March 2019. Although the total number of cases in 2020 (382) was lower than that during 2016–2019 (range = 443–538), the total in 2021 (471) was within this historical range.

**FIGURE:**
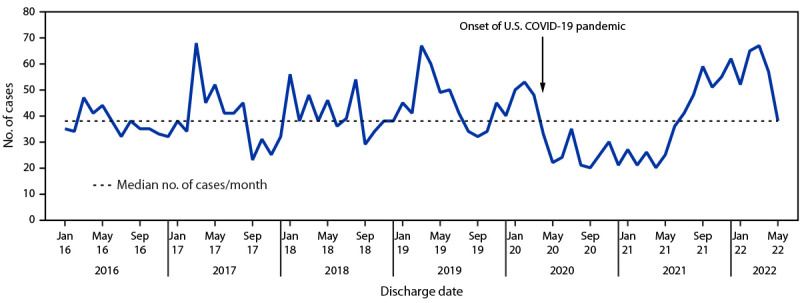
Cases of brain abscess, epidural empyema, or subdural empyema in persons aged ≤18 years — Pediatric Health Information System, United States, January 2016–May 2022* * Data from 40 children’s hospitals.

The median patient age was 8 years (IQR = 1–13 years). Most cases (65.1%) occurred in males; 46.5% of cases were in non-Hispanic White (White), 21.3% in non-Hispanic Black, 20.8% in Hispanic or Latino (Hispanic), 3.3% in non-Hispanic Asian children, and 8.1% in non-Hispanic children of another race. The demographic characteristics of patients remained largely consistent over time, as did markers of severity (e.g., length of hospitalization, in-hospital mortality, and ICU admission) and the percentage of patients with a complex chronic condition (Supplementary Figure 1; https://stacks.cdc.gov/view/cdc/120876) (Supplementary Figure 2; https://stacks.cdc.gov/view/cdc/120877) (Supplementary Figure 3; https://stacks.cdc.gov/view/cdc/120878). The percentage of patients with concurrent COVID-19 varied from 1.3% to 10.9% across quarters (Supplementary Figure 2; https://stacks.cdc.gov/view/cdc/120877) paralleling known COVID-19 waves.**^§^**

## Cases Identified Through CDC’s National Call for Cases

Among the 94 possible cases of pediatric brain abscesses, epidural empyemas, or subdural empyemas reported after CDC’s national call for cases, 81 met the case definition. The median patient age was 11 years (IQR = 6–13 years) (Table). Cases were most frequently reported in males (61.3%) and White (54.5%) children. Forty-five percent of cases occurred in children with underlying health conditions, with asthma (11.5%) being the most common. Among patients, 61.0% had a diagnosis of at least one respiratory infection in the 6 weeks before hospitalization, most commonly sinusitis (26.0%) or COVID-19 (18.2%). Most patients (81.8%) sought outpatient care for the illness episode before hospitalization. Subdural empyema was the most common case presentation (53.1%), followed by brain abscess (37.0%) and epidural empyema (33.3%). Among 71 patients who were no longer hospitalized at the time of reporting, two (2.8%) died. Case report data indicated that streptococcal species were identified in most (92.1%) isolates, commonly *S. intermedius* (41.6%) and *Streptococcus anginosus* (18.4%). Nonstreptococcal species, including 15 unique pathogens, were isolated in 28.9% of cases and in all cases with polymicrobial infections.

**TABLE:** Demographic and clinical characteristics, and microbiology results in patients aged ≤18 years with brain abscess, epidural empyema, or subdural empyema reported to CDC in response to a June 2022 national call for cases (N = 81) — United States, June 2021–August 2022

Characteristic (no. with available information)	No. (%)*
**Demographic**	
**Age, yrs, median (IQR)**	11.0 (6.0–13.0)
**Race or ethnicity (66)**	
White, non-Hispanic	36 (54.5)
Black or African American, non-Hispanic	21 (31.8)
Hispanic or Latino	7 (10.6)
Asian, non-Hispanic	1 (1.5)
Multiple races, non-Hispanic	1 (1.5)
**Sex assigned at birth (80)**	
Male	49 (61.3)
Female	31 (38.8)
**Current gender identity (63)**	
Male	35 (55.6)
Female	28 (44.4)
**Underlying health conditions**	
Any underlying health condition (78)	35 (44.9)
Asthma or reactive airway disease (78)	9 (11.5)
Obesity (78)	4 (5.1)
Seizures/Seizure disorder (78)	3 (3.8)
Congenital heart disease (78)	2 (2.6)
Dental caries or periodontal disease (78)	1 (1.3)
Diabetes mellitus (type 1 or 2) (78)	1 (1.3)
Other underlying condition^†^ (78)	20 (25.6)
**Vaccination information**	
Previous pneumococcal conjugate vaccine (65)	55 (84.6)
Previous SARS-CoV-2 vaccine (59)	15 (25.4)
**Recent medical history**	
**Diagnosis in 6 wks preceding hospitalization**	
**Respiratory infection**^§^ **(77)**	47 (61.0)
**COVID-19 (77)**	14 (18.2)
**Influenza (77)**	1 (1.3)
**Sinusitis (77)**	20 (26.0)
**Upper respiratory infection (77)**	12 (15.6)
**Other respiratory infection^¶^ (77)**	11 (14.3)
Sought prehospitalization care** (77)	63 (81.8)
**Hospitalization**	
Length of stay, days (IQR) (71)	10.0 (6.0–21.0)
**Outcome of hospitalization (80)**	
Discharged to home	59 (73.8)
Discharged to rehab facility	10 (12.5)
Currently hospitalized	9 (11.2)
Deceased	2 (2.5)
**During hospitalization**	
Brain abscess (81)	30 (37.0)
Subdural empyema (81)	43 (53.1)
Epidural empyema (81)	27 (33.3)
Sinusitis (77)	47 (61.0)
Osteomyelitis, including Pott’s puffy tumor (77)	24 (31.2)
Bacterial meningitis (77)	20 (26.0)
Orbital/Periorbital cellulitis (77)	13 (16.9)
Mastoiditis (77)	8 (10.4)
Otitis media (77)	4 (5.2)
Vancomycin received during hospitalization (80)	73 (91.2)
Ceftriaxone received during hospitalization (80)	71 (88.8)
Metronidazole received during hospitalization (80)	65 (81.2)
**Detection of viral respiratory pathogens (52)**	
No pathogens identified	38 (73.1)
Pathogens identified^††^	14 (26.9)
**Microbiology**	
**Pathogens identified (76)**	
*Eikenella corrodens*	5 (6.6)
*Fusobacterium nucleatum*	2 (2.6)
*Parvimonas micra*	5 (6.6)
*Staphylococcus aureus*	4 (5.2)
*Staphylococcus epidermidis*	3 (3.9)
*Streptococcus intermedius*	35 (46.1)
*Streptococcus anginosus*	14 (18.4)
*Streptococcus pneumoniae*	9 (11.8)
*Streptococcus constellatus*	7 (9.2)
*Streptococcus agalactiae*	1 (1.3)
*Streptococcus pasteurianus*	1 (1.3)
Other^§§^	13 (17.1)
**Polymicrobial specimens (76)**	16 (21.1)
**Isolate source (75)**	
Brain abscess	13 (17.3)
Epidural empyema	10 (13.3)
Subdural empyema	17 (22.7)
Blood	10 (13.3)
Cerebrospinal fluid	9 (12.0)
Other^¶¶^	16 (21.3)

**Abbreviations:** ED = emergency department; MRSA = methicillin-resistant *Staphylococcus aureus*; RSV = respiratory syncytial virus; URI = upper respiratory infection.

* Percentages calculated using nonmissing data.

^†^ Other underlying conditions included: Alice in Wonderland syndrome (i.e., dysmetropsia, a rare neurologic disorder characterized by distortions in perception, especially of body image); allergies (seasonal, nonseasonal, and peanut); autism; Castleman disease; cerebral palsy (including spastic quadriplegic); cerebral infarction; chronic nasal congestion; cystic encephalomalacia; epilepsy; frequent nosebleeds; gallstone pancreatitis; global developmental delay; Hashimoto disease; headaches, insomnia; intellectual disability; microcephaly; migraines; MRSA infection; myringotomy tubes; neurofibromatosis type 1; nonaccidental trauma to child; oropharyngeal dysphagia; retinal hemorrhage of both eyes; right spastic hemiparesis; sinusitis; snoring; traumatic brain injury at birth; and Trisomy 21.

^§^ Including COVID-19, influenza, sinusitis, upper respiratory infection, and other respiratory infections.

**^¶^** Other respiratory infections included otitis media (five); parainfluenza (two); cough and fever of unspecified cause (one); URI symptoms but no diagnosis (one); RSV (one); and otitis externa (one).

** In ED, outpatient primary care, or urgent care.

^††^ Viral respiratory pathogens detected during hospitalization included: SARS-CoV-2 (nine), rhinovirus/enterovirus (four), RSV (two), influenza virus (one), adenovirus (one), and parainfluenza virus (one).

^§§^ *Actinomyces* sp. (one), *Clostridium* sp. (one), *Candida parapsilosis* (one), *Cutibacterium acnes* (one), *Haemophilus influenzae* (one), *Klebsiella pneumoniae* (one), *Mycoplasma hominis* (one), *Staphylococcus capitis* (one), *Staphylococcus hominis* (one), *Gemella morbillorum* (one), and unspecified streptococci (three).

^¶¶^ Orbital abscess (two), forehead abscess (one), middle meatus (one), ear aspirate (two), and sinuses (eight).

Antimicrobial susceptibility testing was performed on available *Streptococcus* specimens (two *Streptococcus constellatus* and 16 *S. intermedius*) to identify shared microbiological features among cases. Both *S. constellatus* isolates were intermediately resistant to ampicillin, but susceptible to other antimicrobials tested.^¶^ Nine *S. intermedius* isolates were pan-susceptible. One isolate was resistant to tetracycline only. Four *S. intermedius* isolates displayed a 1.5 *μ*g/mL MIC against vancomycin, slightly above the clinical breakpoint for susceptibility (≤1 *μ*g/mL) and were susceptible to other antimicrobials tested. Two isolates were resistant to multiple antibiotics (erythromycin, clindamycin, and tetracycline) and intermediately resistant to quinupristin-dalfopristin, one of which also displayed a 1.5 *μ*g/mL MIC against vancomycin. Among 15 sequenced *S. intermedius* isolates, the average core genome pairwise distance was approximately 6,200 SNPs, indicating genetic unrelatedness.

## Discussion

Nationally representative hospitalizations during January 2016–May 2022, indicate that the number of pediatric brain abscess, epidural empyema, and subdural empyema cases in 2021 were within historical limits. High case counts in March 2022 were consistent with seasonal peaks in cases observed in March since 2016, but not previously reported. Cases declined in April 2022 and reached the median level by May 2022. Based on these findings, initial reports from clinicians (*1*) are consistent with seasonal fluctuations and a redistribution of cases over time during the COVID-19 pandemic. The finding that *S. intermedius* and *S. constellatus* isolates were largely susceptible to tested antimicrobials is consistent with published reports (*7*,*8*).

Pediatric brain abscess, epidural empyema, and subdural empyema are often preceded by respiratory infection, including in 61.0% of cases reported to CDC, although previous COVID-19 was only reported in 18.2%. The extended period with case numbers below the January 2016–May 2022 median after the onset of the COVID-19 pandemic, followed by a peak in cases during late 2021–early 2022, might reflect altered patterns of respiratory pathogen transmission during the pandemic. Other studies have reported decreased incidences of respiratory and streptococcal infections in children coinciding with the implementation of pandemic-related nonpharmaceutical interventions, which were followed by returns to or rebounds past prepandemic baselines after COVID-19 mitigation measures were relaxed (*9*,*10*). Pediatric brain abscesses and empyemas are serious infections always requiring hospitalization; thus, it is unlikely that the observed trends are the result of altered detection of cases from disruptions to the medical system during the COVID-19 pandemic.

The findings in this report are subject to at least five limitations. First, microbiologic etiology could not be identified from the PHIS hospitalization data. Second, PHIS data reported case numbers, not rates over time. Third, PHIS data from tertiary children’s hospitals might not reflect all hospitals admitting children. Fourth, levels of completeness of case report form variables from CDC’s call for cases varied. Whereas COVID-19 diagnosis before hospitalization was of particular interest, this information might not have been reliably available to medical record abstractors. Finally, selection bias could have occurred in the identification and reporting of cases from CDC’s call for cases. In particular, the phrasing of the call for cases, which highlighted streptococcal species as a potential etiology, might have resulted in underreporting of cases with other etiologies.

Through collaboration with state and local health departments, clinicians, laboratorians, and academic partners, this investigation examined multiyear nationally representative hospitalization data, a large case series with detailed clinical information, and microbiologic features of *Streptococcus* sp. isolated from patients with a diagnosis of brain abscess, epidural empyema, or subdural empyema. After a comparative increase in cases from previous years that began in summer 2021, no evidence of increased case severity, genetic relatedness of streptococcal isolates from different cases, or antimicrobial resistance beyond what is typical for streptococcal species was identified. Case numbers peaked in March 2022, consistent with historical, seasonal fluctuations and declined to baseline in subsequent months. CDC will continue to work with investigation partners to monitor ongoing trends in pediatric brain abscesses and empyemas.

**Summary**

**What is already known about this topic?**

Recent reports have suggested a possible increase in pediatric streptococcal brain abscesses, epidural empyemas, and subdural empyemas.

**What is added by this report?**

After a decline in cases at the onset of the COVID-19 pandemic, cases increased during summer 2021, peaked in March 2022, and then declined to baseline levels. Clinical presentation and microbiological features were stable during this period.

**What are the implications for public health practice?**

Initial reports from clinicians are consistent with seasonal fluctuations and a redistribution of cases over time during the COVID-19 pandemic. No evidence of increased case severity, genetic relatedness of streptococcal isolates from different cases, or increased antimicrobial resistance was identified. Epidemiologic monitoring is continuing.

## Footnotes

* The Pediatric Infectious Diseases Society and the Section of Pediatric Neurosurgeons, a joint section of the American Association of Neurologic Surgeons and Congress of Neurologic Surgeons.

† 45 C.F.R. part 46, 21 C.F.R. part 56; 42 U.S.C. Sect. 241(d); 5 U.S.C. Sect. 552a; 44 U.S.C. Sect. 3501 et seq.

§ https://covid.cdc.gov/covid-data-tracker/#datatracker-home (Accessed August 16, 2022).

¶ Antimicrobials tested include ampicillin, cefotaxime, ceftriaxone, chloramphenicol, clindamycin, daptomycin, erythromycin, levofloxacin, linezolid, meropenem, penicillin, quinupristin-dalfopristin, tetracycline, and vancomycin.
